# Pamidronate Rescue Therapy for Hypercalcemia in a Child With Williams Syndrome

**DOI:** 10.3389/fendo.2018.00240

**Published:** 2018-05-14

**Authors:** Sami A. Sanjad, Bilal Aoun, Halim Yammine, Amina Bassyouni, Pascale E. Karam

**Affiliations:** Department of Pediatrics and Adolescent Medicine, American University of Beirut Medical Center, Beirut, Lebanon

**Keywords:** Williams syndrome, hypercalcemia, pamidronate, nephrocalcinosis, furosemide

## Abstract

A 15-month-old male infant diagnosed with Williams Syndrome (WS) was admitted with severe hypercalcemia and nephrocalcinosis. Intravenous hydration and furosemide failed to yield an appreciable and sustainable fall in serum calcium, while the injection of pamidronate achieved a significant decrease in serum calcium in a short period of time. This bisphosphonate could be considered as a second-line treatment for refractory hypercalcemia in WS.

## Background

Williams Syndrome (WS) is an autosomal dominant multisystem disorder (OMIM 1940505) caused by a deletion of 1.5–1.8 Mb on chromosome 7q11.23, comprising approximately 28 genes. This can be confirmed by molecular cytogenetic analysis using fluorescence *in situ* hybridization (FISH) probe. WS is characterized by facial dysmorphism, cardiovascular abnormalities, developmental delay, and gastrointestinal manifestations in addition to endocrinological disturbances. In children, facial dysmorphism includes depressed nasal bridge, epicanthic folds, long philtrum, small and widely spaced teeth, prominent chin, and mandibular hypoplasia. Congenital cardiac malformations are common and include supravalvular aortic stenosis, supravalvular pulmonary stenosis, pulmonary artery stenosis, and hypertension. Feeding difficulties, failure to thrive, and cognitive impairment are also common features of WS. Endocrine abnormalities, such as diabetes and subclinical hypothyroidism are mainly described in adults while hypercalcemia, occurring in variable frequency and severity, is mostly reported in infants (1). Hypercalcemia is usually associated with hypercalciuria and to a lesser extent nephrocalcinosis (2). Traditional treatment of severe hypercalcemia in children with WS consists of intravenous hydration with isotonic saline, furosemide administration in parallel with dietary calcium, and vitamin D restriction. Some children, however, may be unresponsive to this therapeutic approach and thus warrant alternative treatment. We report a 15-month-old patient with severe hypercalcemia and nephrocalcinosis associated with WS who had an inadequate response to conventional therapeutic modalities. To our knowledge, only four previous reports (1, 3–5) comprising five patients have demonstrated successful treatment of hypercalcemia in WS with intravenous infusions of a biphosphonate compound.

## Case Presentation

A 15-month-old male infant presented for failure to thrive, irritability, and constipation since the age of 6 months. He was the product of full-term pregnancy with no antenatal or perinatal complications. Physical exam revealed a small, undernourished baby with a peculiar facies. His weight was 6.3 kg; length 74 cm; and head circumference 42 cm, all significantly below the third centile for age. The rest of the physical exam was unremarkable. Laboratory tests revealed hypercalcemia at 17.5 mg/dl (normal: 8.4–10.6 mg/dl), normal serum phosphorus at 4.2 mg/dl (normal: 2.4–4.9 mg/dl), and high urinary calcium/creatinine ratio (UCa/UCr) at 1.1 (normal value up to 0.5) (6) with normal serum 25-hydroxyvitamin D (25 OH-vit D) at 32.3 ng/ml (normal: 20–60 ng/ml). Renal ultrasound showed grade III bilateral medullary nephrocalcinosis and echocardiography detected mild supravalvular aortic stenosis. The diagnosis of Williams’s syndrome was then confirmed by FISH probe analysis which revealed a deletion at 7q11.23 encompassing the elastin gene. Hypercalcemia was treated by the traditional approach with intravenous hydration and furosemide infusion (1 mg/kg). Serum calcium decreased to 12 mg/dl and the patient was discharged on calcium-restricted diet. Ten days later, however, serum calcium rose to 16.1 mg/dl. The same intravenous treatment regimen was used and serum calcium decreased to 11.6 mg/dl for 48 h, only to rise to 17 mg/dl following discontinuation of furosemide. Serum intact parathyroid hormone (PTH) was suppressed at 1.5 pg/ml (normal: 10–65 pg/ml). He received, then, an infusion of 1 mg/kg pamidronate over 6 h with no side effects observed. Two days post-infusion, serum calcium dropped to 11.8 mg/dl and UCa/UCr to 0.6. Patient was discharged home on calcium unrestricted diet. One month later, he received a second intravenous infusion of 1 mg/kg pamidronate for recurrent hypercalcemia up to 14.1 mg/dl, which decreased serum calcium again to 9.9 mg/dl. Subsequently, the patient was kept on a calcium unrestricted diet and maintained normal serum calcium level of 9.5 mg/dl, UCa/UCr of 0.12 as well as 25 OH-vit D of 30 ng/ml, at 5 years of follow-up. At 6 years of age, his growth parameters had improved considerably. He is currently at the fifth percentile for height and between fifth and tenth percentile for weight. Renal ultrasound showed stable grade III bilateral medullary nephrocalcinosis.

## Discussion

The reported incidence of at least one episode of hypercalcemia in WS varies between less than 5 and 50% (6, 7). This wide discrepancy may be due to different study designs and also to the fact that hypercalcemia is frequently transient in infancy and may have been missed in some of the series reporting a very low incidence. Hypercalcemia is usually associated with hypercalciuria in 15–40% of WS cases (3). It often disappears after the age of one year, but may return during puberty and at times may be refractory to traditional therapy. Nephrocalcinosis in WS is thought to be rare occurring in 5% of WS patients (8). When discovered, hypercalcemia in WS is often treated conservatively with intravenous saline and furosemide with careful attention to replace urinary electrolyte losses and avoid hypokalemia; however, no specific guidelines for conventional therapy have been recommended. To our knowledge, only five previous WS patients with refractory hypercalcemia were successfully treated with bisphosphonate infusions (Table 1). To date, no approved regimens for pamidronate use in WS have been described in the literature (3). Moreover, the use of bisphosphonates in the pediatric population still lacks Food and Drug Administration approval. Use of this class of medication, not only in WS, but also in other conditions causing hypercalcemia, should be considered as a second-line therapy taking into consideration the benefits versus the side effects associated with their acute and long-term use (9). Hypocalcemia and flu-like syndrome may occur acutely, while drug-related osteopetrosis (10) was reported with chronic use of cumulative doses of pamidronate. Potential side effects on growth (11) may also be concerning; however, patients treated on a long-term basis with pamidronate infusions for conditions other than WS, achieved normal linear growth (12).

**Table 1:** Williams syndrome patients treated with pamidronate.

	Patient 1 (3)	Patient 2 (1)	Patient 3 (1)	Patient 4 (4)	Patient 5 (5)	Patient 6^a^
Age (months)/gender	14/male	11/male	1/male	17/female	36/male	15/male
Clinical presentation	FTT, PMD	Decreased appetite	Irritability	FTT, PMD	FTT, PMD	FTT
	Dysmorphism	Irritability		Constipation	Constipation	Dysmorphism
	Dehydration	Hypotonia		Polyuria		Constipation
		Constipation		Polydipsia		PMD
Echocardiography	Normal	Not recorded	PS, SVAS	Mild ASD	Not recorded	SVAS
Renal ultrasound	Not recorded	NC	NC	NC grade I	NC	NC grade III

**Initial laboratory tests**						
Calcium (mg/dl)	13	17.7	13.5	16	14.5	17.5
Phosphorus (mg/dl)	4	4.7	5.1	3.7	6.2	4
Intact PTH pg/ml	<10	5	4	6.7	2.5	1.5
25 OH-vit D ng/ml	50	25	30	24.6	21.4	32
1,25 OH vit D pg/ml	48	10	13	–		–
Urine calcium/creatinine	0.81	0.75	1.3	1.4	2.4	1

**Treatment**						
Initial	Pamidronate	IV furosemide	Oral furosemide	IV furosemide	IV furosemide	IV furosemide
		Low calcium diet calcitonin		Low calcium diet	Low calcium diet	Low calcium diet
Pamidronate dosage	1 mg/kg over 4 h	1 mg/kg over 6 h with oral acetaminophen	1 mg/kg over 6 h	1 mg/kg over 6 h	1 mg/kg over 6 h	1 mg/kg over 6 h
				0.5 mg/kg after 24 h		
Total number of pamidronate doses	1	1	4	2	1	2
Follow-up duration (months)	12	13	28	7	12	53

*FTT, failure to thrive; PMD, psychomotor delay; PS, pulmonary stenosis; SVAS, supra valvular aortic stenosis; ASD, auricular septal defect; NC, nephrocalcinosis; PTH, parathyroid hormone; Ca/Creat, calcium/creatinine ratio; IV, intravenous*.

*^a^Current report*.

Although the mechanism is not fully understood, severe hypercalcemia may be seen occasionally in patients with WS. The hypercalcemia may be refractory to conventional treatment as was the case with our patient. Even when mild, the insidious effects of hypercalcemia may be devastating and should be treated promptly and effectively. It has been suggested that the increase in calcium levels seen in WS patients may be due to problems related to vitamin D metabolism or calcitonin gene expression, while others have postulated that calcium levels rise due to increased release from bone (1, 13, 14). The efficacy of pamidronate and other bisphosphonates in reducing calcium levels supports the theory that the hypercalcemia in WS is probably due to calcium release from bone. However, the finding of normal alkaline phosphatase levels in patients with WS argues against that theory. Additional studies, perhaps including bone biopsy to investigate the state of bone turnover in WS, may be necessary to explore the etiology of hypercalcemia in this condition. Hyper-reabsorption of calcium from the gastrointestinal tract and renal tubular cells has been suggested as a possible mechanism underlying hypercalcemia in patients with WS (1, 15). Long-term calcium dietary restriction, however, was not necessary in 3 out of the 6 patients who were successfully treated with pamidronate.

## Conclusion

Regardless of the pathophysiology, hypercalcemia remains a major morbidity factor and should not be overlooked in anyone suspected of having WS. In patients with severe and refractory hypercalcemia, pamidronate at a dose of 1 mg/kg seems to be an alternative, safe, and effective treatment. The paucity of cases of WS treated with pamidronate and the lack of evidence-based treatment guidelines for such patients may open the way for future multi-center studies including a larger number of patients.

## Bioethics

Written informed consent was obtained from the parents of the participant for the publication of this case report.

## Ethics Statement

This study was carried out in accordance with the recommendations of the institutional review board at the American University of Beirut, Lebanon.

## Author Contributions

BA and SS equally contributed to this manuscript. BA, SS, and PK contributed to the conception and design of the study. HY and AB collected clinical data. BA, SS, and HY wrote the first draft of the manuscript. BA, SS, and PK wrote sections of the manuscript. PK contributed to manuscript revision. All authors read and approved the submitted version.

## Conflict of Interest Statement

The authors declare that the research was conducted in the absence of any commercial or financial relationships that could be construed as a potential conflict of interest.
